# Comparison of dual antiplatelet therapies after coronary endarterectomy combined with coronary artery bypass grafting: a cohort study

**DOI:** 10.1186/s13019-020-01205-z

**Published:** 2020-06-29

**Authors:** Hua Yan, Xieraili Tiemuerniyazi, Yangwu Song, Fei Xu, Wei Feng

**Affiliations:** Department of Cardiovascular Surgery, Fuwai Hospital, National Center for Cardiovascular Diseases, Chinese Academy of Medical Sciences and Peking Union Medical College, Beilishi Road No. 167, Xicheng District, Beijing, 100037 China

**Keywords:** Coronary endarterectomy, Coronary artery bypass grafting, Dual antiplatelet therapy, Ticagrelor, Clopidogrel

## Abstract

**Background:**

Coronary endarterectomy (CE) combined with coronary artery bypass grafting (CABG) can be the only option for complete revascularization in some patients with diffuse coronary artery disease. Unfortunately, CE can cause the lack of endothelium, resulting in increased risk of thrombotic events. Therefore, antithrombotic therapy is very important after surgery. However, there’s no consistent protocol exists till now. The aim of this study was to compare the effectiveness and safety of dual antiplatelet therapies (DAPT) including aspirin plus clopidogrel (AC) or ticagrelor (AT) after CE + CABG.

**Method:**

A total of 137 continuous patients (mean age 60.0 ± 9.0 years) underwent CE + CABG from January 2016 to July 2018 in our center, and patients who received dual antiplatelet therapy (DAPT) after surgery (*n* = 121) were included in this study. All of the patients received aspirin 100 mg daily therapy after surgery, and 67 of the patients received extra clopidogrel 75 mg (AC) daily therapy, whereas 54 received extra ticagrelor 90 mg (AT) twice daily. All patients continued aspirin monotherapy after 1 year. Occurrence of ischemic events and bleeding events between two groups were compared. Kaplan-Meier survival was used to compare freedom from major adverse cardiovascular and cerebrovascular events (MACCE) between two groups, and log-rank test was used to confirm statistical difference.

**Results:**

Follow-up was completed by 99.2%, and median follow-up time was 30.0(22.5, 35.2) months. No operative death was observed, while perioperative myocardial infarction was observed in 2(1.7%) patients (AC 1.5% vs. AT 1.9%, p = ns). One patient in AC group suffered from cardiac tamponade. During the follow-up period, no death was observed. Ischemic events including nonfatal myocardial infarction, repeat revascularization and ischemic stroke were observed in 6(5.0%) patients (AC 4.5% vs. AT 5.6%, p = ns). Overt bleeding had occurred in 3(2.5%) patients (AC 3.0% vs. AT 1.9%, p = ns). Kaplan-Meier analysis indicated that MACCE-free survival of the two groups at 3 years was 97.0% in the AC group versus 94.1% in the AT group (p = ns).

**Conclusion:**

In patients undergoing CE + CABG, DAPT therapy can be effective and safe with comparable results between AC and AT therapy in terms of ischemic and bleeding events. Further studies are needed.

## Background

Coronary endarterectomy (CE) for the treatment of advanced coronary artery disease can be traced back to 1950s [1]. Before long, it became an adjunctive way to coronary artery bypass grafting (CABG) in patients with diffuse coronary artery disease that could not be achieved complete revascularization by CABG or coronary stenting alone. Unfortunately, early results from clinical studies were frustrating [2]. In contrast, studies reported improved clinical outcomes after CE + CABG during last two decades [3, 4], and this might be attributed to the improvement in operative techniques and postoperative antithrombotic therapy. However, controversies still exist.

Thrombosis is the major cause of early graft occlusion, and this can lead to poor patient prognosis [5]. Activation of platelet function and even coagulation cascade can occur due to the traumatic stress, blood loss and cardiopulmonary bypass during CABG, and this can last up to more than 1 month [6–8], making antithrombotic treatment particularly important. One of the fatal shortcomings of CE is the loss of intima, which causes direct exposure of sub-endothelial tissue to blood flow, resulting in increased platelet aggregation and activation of coagulation cascade [9]. In other words, there might be more chances of thrombosis after CE + CABG compared to CABG alone. Therefore, more active postoperative antithrombotic therapy might be required after CE + CABG.

Clinical guidelines recommend that patients should receive dual antiplatelet therapy (DAPT) for 12 months after CABG [10, 11]. Aspirin plus clopidogrel therapy is the conventional DAPT protocol. However, due to the diversity of drug reactions, sufficient platelet inhibition cannot be achieved in 10 to 63% of patients with clopidogrel [12, 13]. Ticagrelor, a new P2Y12 receptor antagonist, can achieve stronger and faster platelet inhibition with safe clinical outcomes, and there’s no drug reaction diversity when compared with clopidogrel [14–16]. Currently clinical guideline from the European Society of Cardiology recommended both clopidogrel and ticagrelor for DAPT in patients with coronary artery disease [11]. However, there’s no consistent antiplatelet therapy protocol after CE + CABG. Several studies existed. Marco Russo and colleagues [17] reported that no significant difference was observed between single antiplatelet therapy with aspirin and DAPT with aspirin plus clopidogrel after CE + CABG, although the study was limited with small sample size. Several studies involved anticoagulation or antiplatelet therapy after CE + CABG [4], but without comparing different protocols. Therefore, more evidence on antithrombotic therapy after CE + CABG is needed.

Since it has more rapid onset and offset of action, and there’s no drug reaction diversity, ticagrelor might be better than clopidogrel in patients who underwent CE + CABG. This study was aimed to compare the efficacy and safety outcomes of different DAPT protocols, aspirin plus clopidogrel (AC) versus aspirin plus ticagrelor (AT) therapy, after CE + CABG, as well as to evaluate major adverse cardiovascular and cerebrovascular events (MACCE) free survival of the patients in two groups.

## Patients and method

### Patients and study design

**Fig. 1:**
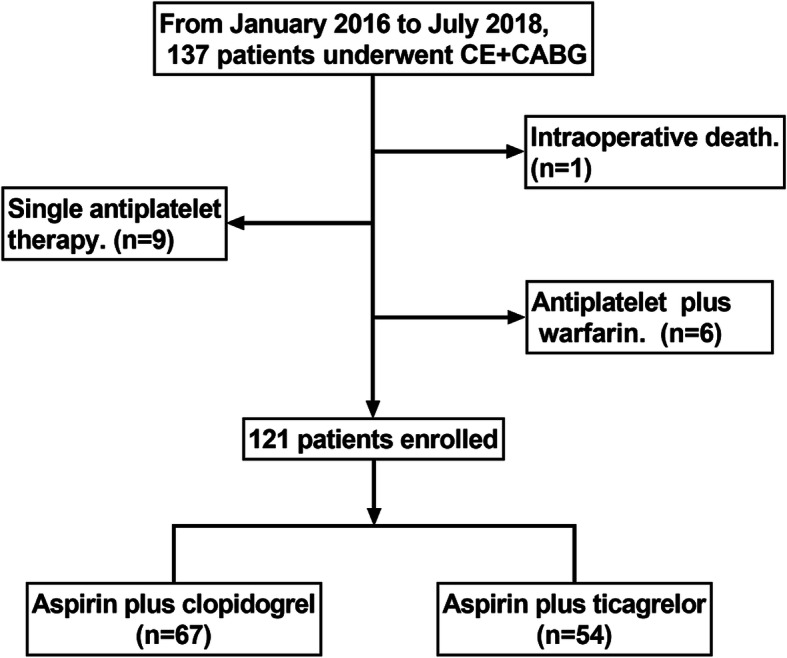
Flow chart of the patient enrollment process. A total of 137 continuous patients underwent CE + CABG during the study period. Sixteen patients were excluded for specific reasons showed in the figure, and 121 of the patients enrolled. (CE, coronary endarterectomy; CABG, coronary artery bypass grafting)

This was a cohort study from our center. A total of 137 continuous patients underwent CE + CABG from January 2016 to July 2018 in our center. One patient died intraoperatively, and 15 patients received single antiplatelet therapy or antiplatelet plus anticoagulation therapy. Therefore, 121 of the patients, who received DAPT only, were finally enrolled. Figure 1 showed the patient enrollment process. Patients were divided into AC and AT groups according to their postoperative DAPT protocol. All of the patients received aspirin 100 mg daily therapy, and patients in the AC group received extra clopidogrel 75 mg daily therapy, while patients in the AT group received extra ticagrelor 90 mg twice daily therapy after surgery. All patients continued single antiplatelet therapy with aspirin after 12 months. The institutional review board at Fuwai Hospital approved the usage of clinical data, and waived individual informed consent for this study.

### Operative techniques

Each patient received CE at least on one site, followed by CABG. The indications for CE included: diffuse coronary artery disease that cannot completely revascularized by CABG alone due to long segment coronary artery occlusion or lumen less than 1 mm, or severe calcification, which was determined by preoperative coronary angiography or intraoperative exploration. Ultimate decision for CE was made during the operation. CE was performed by experienced cardiac surgeons by on-pump or off-pump path to achieve complete revascularization before CABG. Closed endarterectomy was preferred in most of the patients, and several patients received patch angioplasty after CE.

### Data collection, follow-up and definitions

Baseline and perioperative characteristics of the patients were collected from reviewing the inpatient medical records, while follow-up was achieved mainly by telephone interview. Primary endpoint was the occurrence of ischemic events defined as the composite of nonfatal myocardial infarction, repeat revascularization, and nonfatal ischemic stroke. Secondary endpoint was the occurrence of bleeding events, which was defined according to Bleeding Academic Research Consortium Definition for Bleeding definition [18]. Overt bleeding events, which required medical therapy, or cessation of antiplatelet therapy, or threatened the life of patients (type2 to type5), were mainly collected during the follow-up.

Perioperative myocardial infarction was defined according to the fourth universal definition of myocardial infarction [19]. MACCE was defined as the composite of all-cause death, nonfatal myocardial infarction, unplanned repeat coronary revascularization and stroke.

### Statistical analysis

Continuous variables were expressed as mean ± standard deviation if normally distributed and skewed continuous variables were expressed as the median with the interquartile range. Categorical variables were expressed as frequencies followed by percentages. Normally distributed continuous variables were compared by paired or non-paired Student t-test, and skewed continuous variables were compared using the Wilcoxon’s rank-sum test, while categorical variables were compared by Chi-square statistics or Fisher exact test, as appropriate. Cumulative survival and the MACCE-free survival rates were estimated using the Kaplan-Meier method and compared by log-rank test. Differences with *p* values of less than 0.05 were considered statistically significant. Statistical analyses were performed using STATA (15.0) (StataCorp, College Station, TX, USA).

## Results

### Patient characteristics

Mean age was 60.0 ± 9.0 years, and 94(77.7%) were male. History of prior myocardial infarction differed between AC group and AT group (*p* = 0.043). Comparison of preoperative baseline characteristics between AC group and AT group were summarized in Table 1.

**Table 1:** Baseline characteristics of the patients

Characteristics	AC group(*n* = 67) No. (%), Mean ± SD, Median (range)	AT group(*n* = 54) No. (%), Mean ± SD, Median (range)	*p* value
Age	61.4 ± 8.5	58.2 ± 9.3	0.055
BMI, kg/m^2^	25.5 (23.5, 27.5)	25.7 (24.1, 28.0)	0.257
Sex, male	50 (74.6%)	44 (81.5%)	0.368
Prior PCI	10 (14.9%)	19 (16.7%)	0.794
Atrial fibrillation	2 (3.0%)	2 (3.7%)	–
Hypertension	45 (67.2%)	34 (64.8%)	0.786
Dyslipidemia	56 (83.6%)	50 (92.6%)	0.135
Diabetes mellitus	30 (44.8%)	21 (38.9%)	0.514
Cerebrovascular event	11 (16.4%)	11 (20.4%)	0.575
Chronic kidney failure	2 (3.0%)	1 (1.9%)	–
Smoking	39 (58.2%)	40 (74.1%)	0.068
Prior cardiac surgery	1 (1.5%)	1 (1.9%)	–
Prior myocardial infarction	32 (47.8%)	16 (29.6%)	0.043^a^
Triple-vessel disease	49 (73.1%)	32 (59.3%)	0.107
NYHA class III or IV	29 (43.3%)	31 (57.4%)	0.122
Preoperative EF, %	62.0 (57.0–66.0)	60.0 (56.0–65.0)	0.449

*BMI* body mass index, *EF* ejection fraction, *No.* number, *NYHA* New York Heart Association, *PCI* percutaneous coronary intervention, *SD* standard deviation

^a^ Statistically significant

Most of the patients underwent off-pump surgery, and all of the patients received internal mammary artery (IMA, mostly left IMA) graft to left anterior descending artery (LAD) in both groups. Most of the patients received CE on LAD (38.8%) or right coronary artery (RCA, 51.2%). Several patients also received CE on other sites, including left circumflex artery (7.4%) and other coronary arteries (5.8%) like diagonal branches or intermediate artery. CE sites and other intraoperative characteristics of the patients were comparable between two groups, as shown in Table 2.

**Table 2:** Intraoperative and postoperative characteristics

Variables	AC group(*n* = 67) No. (%), Mean ± SD, Median (range)	AT group(*n* = 54) No. (%), Mean ± SD, Median (range)	*p* value
On-pump	27 (40.3%)	13 (24.1%)	0.059
No. of distal anastomosis	3.5 ± 0.8	3.4 ± 0.9	0.391
Usage of IMA to LAD	67 (100%)	54 (100%)	–
Coronary endarterectomy			
LAD	26 (38.8%)	21 (38.9%)	0.993
Right coronary artery	33 (49.3%)	26 (48.2%)	0.904
Left circumflex artery	4 (6.0%)	5 (9.3%)	0.493
Other (intermediate, diagonal)	4 (6.0%)	5 (5.6%)	–
ICU stay	44.0 (19,70)	24.5 (21.0,69.0)	0.365
Transfusion	9 (13.4%)	3 (5.7%)	0.150
Postoperative EF, %	60.0 (58.0,63.0)	60.0 (55.0,63.0)	0.330
Cardiac tamponade	1 (1.5%)	0	–
Perioperative MI	1 (1.5%)	1 (1.9%)	–
New onset atrial fibrillation	11 (16.4%)	9 (16.7%)	0.971
Sternal wound infection	1 (1.5%)	0	–

*AC* aspirin plus clopidogrel, *AT* aspirin plus ticagrelor, *EF* ejection fraction, *ICU* intensive care unit, *IMA* internal mammary artery, *MI* myocardial infarction, *No.* number, *SD* standard deviation

### Early postoperative results

Mean ICU stay were 44.0(19, 70) hours in AC group and 24.5(21.0, 69.0) hours in AT group (*p* = 0.365). There was no operative death. Perioperative myocardial infarction was observed in 2(1.7%) patients (AC 1.5% vs. AT 1.9%), while postoperative new-onset atrial fibrillation was seen in 20(16.5%) patients (AC 16.4% vs. AT 16.7%, *p* = 0.971). One patient in AC group suffered from cardiac tamponade at early stage. Early postoperative results were summarized in Table 2.

### Follow-up results

Follow-up was completed by 99.2%, where one patient was lost to contact, and median follow-up time was 30.0(22.5, 35.2) months. During the follow-up period, no death was observed. Ischemic events were observed in 6(5.0%) patients without significant difference between two groups (AC 4.5% vs. AT 5.6%). Ischemic events included nonfatal myocardial infarction (AC 3.0% vs. AT 3.7%), ischemic stroke (AC 1.5% vs. AT 0), and repeat revascularization (AC 0 vs. AT 1.9%). Bleeding events were observed in 3(2.5%) patients (AC 3.0% vs. AT 1.9%), including cardiac tamponade (AC 1.5% vs. AT 0), hemorrhagic stroke (AC 0 vs. AT 1.9%) and gastrointestinal bleeding (AC 1.5% vs. AT 0), and there was no significant difference between two groups as well. Follow-up outcomes were summarized in Table 3.

**Table 3:** Follow-up outcomes

Variables	AC group (*n* = 67)	AT group (*n* = 54)	*p* value
All-cause death	0	0	–
Ischemic events	3 (4.5%)	3 (5.6%)	–
Myocardial infarction	2 (3.0%)	2 (3.7%)	
Ischemic stroke	1 (1.5%)	0	
Repeat revascularization	0	1 (1.9%)	
Bleeding events	2 (3.0%)	1 (1.9%)	–
Cardiac tamponade	1 (1.5%)	0	
Hemorrhagic stroke	0	1 (1.9%)	
Gastrointestinal bleeding	1 (1.5%)	0	
MACCE	2 (3.0%)	3 (5.6%)	0.514

*AC* aspirin plus clopidogrel, *AT* aspirin plus ticagrelor, *MACCE* major cardiac and cerebrovascular events, *No.* number, *SD* standard deviation

**Fig. 2:**
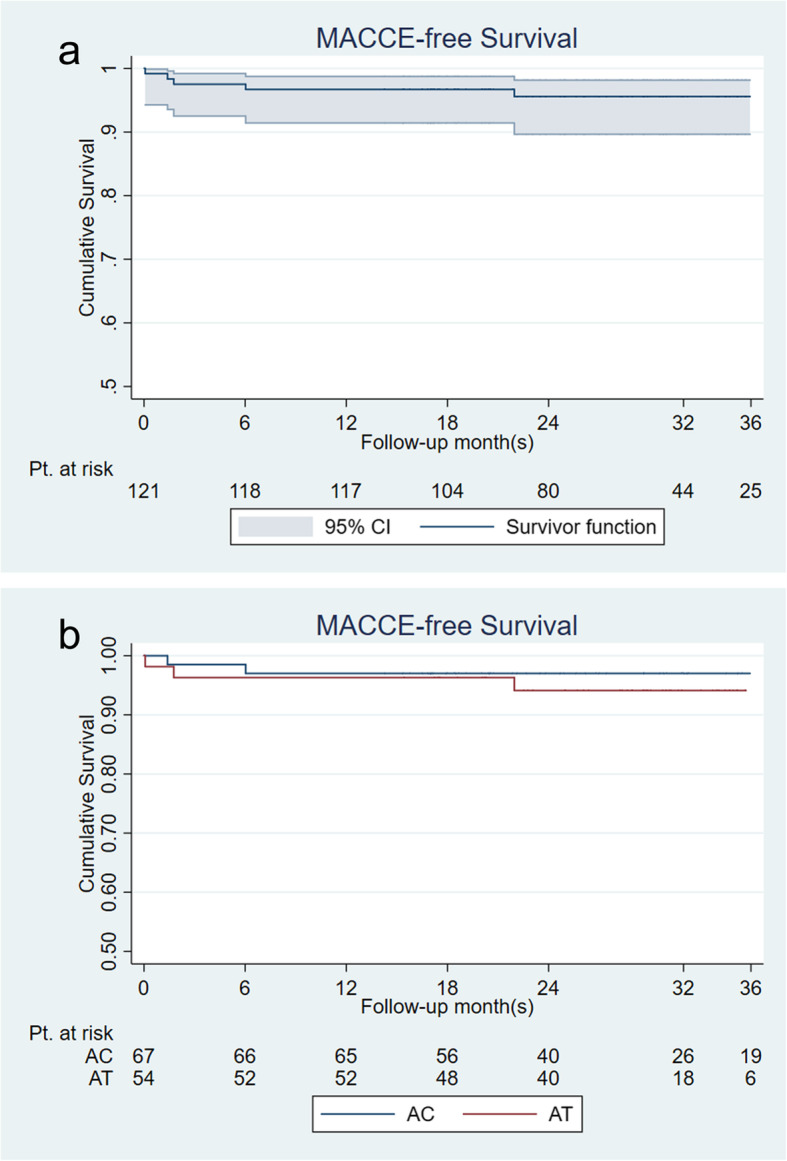
MACCE-free survival of the patients. **a** showed the overall MACCE-free survival of all patients; **b** showed the comparison of MACCE-free survival between AC group and AT group. (AC, aspirin plus clopidogrel; AT, aspirin plus ticagrelor; CI, confidence interval; MACCE, major adverse cardiovascular and cerebrovascular events; Pt., patients)

Kaplan-Meier survival function was performed to analyze MACCE-free survival of the patients after CE + CABG. Incidence of MACCE was 4.1% in total patients during the follow-up, and Fig. 2(A) showed the overall MACCE-free survival. MACCE-free survival of the two groups at 3 years was 97.0% in the AC group versus 94.1% in the AT group without difference in the log-rank test (*p* = 0.514). Figure 2(B) showed the Kaplan-Meier survival curve of the two groups.

## Discussion

In this study, we compared the efficacy and safety of two different DAPT protocols, AC versus AT, after CE + CABG. We observed there’s no significant difference concerning ischemic and bleeding events, as well as overall MACCE-free survival between the two groups during mid-term follow-up.

Complete revascularization is very important in patients with coronary artery disease, where satisfactory coronary revascularization can be achieved by CABG alone or coronary stenting in most cases. However, it is a challenge for surgeons to achieve complete revascularization in those with diffuse coronary artery disease by conventional techniques. In these cases, CE had its priority when used adjunctively to CABG. Unlike early study results, researchers reported improved early and late outcomes after CE + CABG in several individual studies during last two decades [3, 4, 20], although most of the studies were restricted with observational design. Undoubtedly, these results might be attributed to the improvement in operative techniques and postoperative management.

Antithrombotic treatment is one of the most important treatments to improve graft patency and clinical outcomes of patients after coronary revascularization. Loss of coronary intima is one of the disadvantages caused by CE, which in turns leads to the direct exposure of sub-intimal tissues to blood flow [9] and release of tissue factor. This can be followed by platelet activation and aggregation, resulting in increased risk of thrombosis. Therefore, patients who underwent CE + CABG might need more strict antiplatelet therapy compared to CABG alone or stenting.

Clinical guidelines recommended DAPT after CABG alone [10, 11], including aspirin plus clopidogrel or ticagrelor. Different from clopidogrel, ticagrelor is a direct-acting and reversible P2Y12 receptor antagonist with faster and more consistent antiplatelet inhibition effect [21, 22]. Furthermore, both prototype and metabolites of ticagrelor have biological effect [23], which means its effect is stronger and not limited by CYP2C19 gene polymorphism. Several clinical trials also reported stronger platelet inhibition after ticagrelor than clopidogrel [14–16]. However, there’s no guideline recommendation with respect to antithrombotic therapy after CE + CABG. There’re limited studies that focused on antiplatelet therapy after CE + CABG. In one retrospective study, Russo and colleagues [17] reported compared results in single versus dual antiplatelet therapy after CE + CABG, but this study was restricted with small sample size. Others involved antithrombotic treatment protocol in their studies, but not focusing on the comparison of antiplatelet therapy [4]. Therefore, more evidence is needed.

Study results indicated good efficacy outcome after aspirin with ticagrelor therapy in patients after CABG [15]. In this study, we observed that composite of ischemic events including nonfatal myocardial infarction, repeat revascularization and ischemic stroke, was comparable between AC group and AT group. This was consistent with the result of a meta-analysis [24], although the study population was from patients with acute coronary syndrome. What cannot be ignored is that sample size of our study was not large enough, and this might have limited the power of test.

In addition to efficacy outcome, safety outcome of antiplatelet drugs is also important. Researchers reported ticagrelor was associated with higher risk of major bleeding when compared with clopidogrel in East Asian patients with acute coronary syndrome in a subgroup analysis of PLATO trial [25]. In this study, bleeding events that required cessation of antiplatelet drugs or medical intervention, or that was life-threatening, was investigated. Two patients in AC group suffered from bleeding events including cardiac tamponade and gastrointestinal bleeding, while one patient in AT group suffered from hemorrhagic stroke. No difference was observed regarding bleeding events between AC group and AT group. However, it is worth mentioning that minor bleeding events that did not require medical consultation or cessation of drugs, such as subcutaneous petechiae and nasal bleeding, were non considered into account in this study. We also observed that there was no significant difference in the rate of MACCE occurrence between two groups. More large-sample sized studies are needed to confirm these results.

Our study has several limitations. Firstly, this was an observational study, and the bias caused by the study design could not be avoided. Secondly, the sample size was limited, which might reduce the power of tests. Last but by no means least, we collected clinically overt bleeding events, which was defined elsewhere, when comparing the drug safety events between two groups. This might compromise the comparison of secondary endpoint.

## Conclusion

In patients undergoing CE + CABG, DAPT therapy can be effective and safe with comparable results between AC and AT therapy in terms of ischemic and bleeding events. Further studies are needed.

## Data Availability

The data used to support the findings of this study are available from the corresponding author upon request.

## Abbreviations

AC: aspirin plus clopidogrel
AT: aspirin plus ticagrelor
CABG: coronary artery bypass grafting
CE: coronary endarterectomy
DAPT: dual antiplatelet therapy
IMA: internal mammary artery
LAD: left anterior descending artery
MACCE: major adverse cardiovascular and cerebrovascular events
RCA: right coronary artery
